# The benefits of psychosocial interventions for cancer patients undergoing radiotherapy

**DOI:** 10.1186/1477-7525-11-121

**Published:** 2013-07-17

**Authors:** Zhen Guo, Hua-ying Tang, Hao Li, Sheng-kui Tan, Kai-hua Feng, Yin-chun Huang, Qing Bu, Wei Jiang

**Affiliations:** 1 Department of Radiation Oncology, Guilin Medical College Affiliated Hospital, 15 Lequn Road, Guilin, People’s Republic of China; 2 School of Nursing, Guilin Medical College, 109 Huanchen Road North, Guilin, People’s Republic of China; 3 Department of Psychiatry, Guilin Medical College Affiliated Hospital, 15 Lequn Road, Guilin, People’s Republic of China; 4 School of Public Health, Guilin Medical College, 109 Huanchen Road North, Guilin, People’s Republic of China; 5 Department of Medical Oncology, Guilin Medical College Affiliated Hospital, 15 Lequn Road, Guilin, People’s Republic of China

**Keywords:** Cancer, Radiation oncology, Psychosocial intervention, Anxiety, Depression, Quality of life

## Abstract

**Background:**

Many patients with cancer experience depression and anxiety, and an associated decrease in quality of life (QOL) during radiation therapy (RT). The main objective of the study was to determine the benefits of psychosocial interventions for cancer patients who received RT.

**Methods:**

Patients with cancer (n = 178) who agreed to participate in the study were randomized to the intervention arm (n = 89) or the control arm (n = 89). Patients in the intervention group received psychosocial care during RT, whereas the control group received RT only. The benefits of the intervention were evaluated using the Zung Self-rating Depression Scale (SDS) to measure depression, the Self-rating Anxiety Scale (SAS) to assess anxiety, and the European Organization for Research and Treatment of Cancer Quality of Life Questionnaire-Core 30 (EORTC QLQ-C30) to survey health-related QOL. The association between intervention and survival was also assessed.

**Results:**

Patients randomly assigned to the intervention arm showed significant improvements on symptoms of depression (*p* < 0.05) and anxiety (*p* < 0.05), health-related QOL (*p* < 0.05) (i.e. better global health status, and physical and emotional functioning, and less insomnia) when compared with controls. In the subset analysis, female patients, those that received high dose irradiation, and those that underwent adjuvant chemotherapy could benefit more from psychosocial intervention. There was no difference between the two groups in disease-free survival (DFS) (2-year DFS 79.8% in the intervention arm and 76.4% in the control arm; *p* = 0.527) and overall survival (OS) (2-year OS 83.1% in the intervention arm and 84.3% in the control arm; *p* = 0.925)

**Conclusions:**

Psychosocial intervention is a cost-effective approach that can improve a patient’s mood and QOL both during and after RT. However, the intervention was not found to reduce the risk of cancer recurrence and death.

**Trial registration:**

ChiCTR-TRC-12002438

## Background

During the course of cancer treatment, about two-thirds of patients will undergo radiation therapy (RT) as an essential component of a treatment program aimed at curing the disease, prolonging life or palliating symptoms [1] . However, RT often has a strong negative impact on cancer patients: it commonly leads to long-term physical effects(e.g. pain, and decreased physical functioning) and emotional distress (e.g. anxiety and depression) [2]. Various studies have demonstrated that anxiety and depression are important and prevalent problems [3,4] that affect QOL in patients with cancer [5-8]; they also reduce compliance with treatment and prolong hospitalization [9,10], and can compound the physical consequences of the disease [11,12]. Studies have shown that about 41% of tumor patients need professional psycho-oncological support [13], but less than 10% of patients are referred for psychosocial intervention in clinical practice daily [14].

Although a psychological burden associated with cancer is common, it is not inevitable. Psychosocial interventions have been shown to be effective in reducing distress in cancer patients. For example, Osborn analyzed 15 randomized controlled trials that investigated the effects of psychosocial intervention on commonly reported problems such as depression, anxiety, pain, physical functioning, and QOL in adult cancer survivors, and reported that individual interventions were more effective than a control group. The interventions were found to reduce emotional distress and improving QOL in those surviving cancer [15]. Similarly, a recent meta-analysis by Sheinfeld and colleagues reviewed 37 papers (which included 4199 participants), with pain severity and interference as primary outcome measures. The analysis found that psychosocial interventions had medium-size effects on both pain severity and interference, and suggested that such interventions should be a part of a multimodal approach to the management of pain in patients with cancer [16]. Further benefits of psychosocial interventions were found in extensive studies. Such psychological interventions were regarded as an inexpensive way to reduce psychological distress [17] and possibly to improve immune system functioning [18,19] and prolong survival in patients with cancer [20].

However, few studies have compared emotional state and QOL in those receiving psychosocial intervention and a control group in cancer populations undergoing RT. The goal of this study was to adapt a randomized parallel control design to investigate whether a psychosocial intervention before and during RT could reduce emotional distress and improve treatment outcomes, including measures of QOL in patients newly diagnosed with cancer.

## Methods

### Design

**Figure 1:**
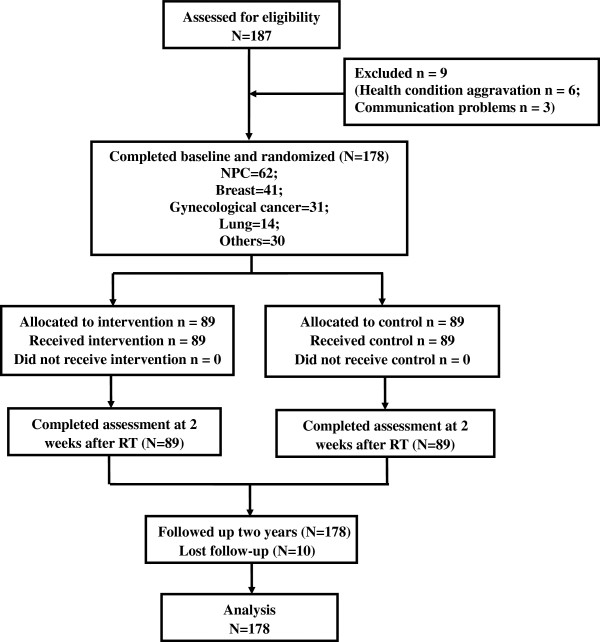
Patient flow and follow-up in the study.

The study will evaluate the benefits of psychosocial interventions for cancer patients within the period of RT through a two-armed randomized controlled trial (Clinical trial registration: ChiCTR-TRC-12002438; http://apps.who.int/trialsearch/ Trial.aspx? TrialID = ChiCTR-TRC-12002438.) Participants were randomized to one of two groups: RT alone (control group) or a psychosocial intervention plus RT. The status of patients was measured during the period between confirmation of the diagnosis and the start of RT, and again two weeks after the completion of RT. All patients were followed up over 2 years (Figure 1). The intervention was carried out by specially trained conductors. All data were collected through self-administered questionnaires. Ethics approval had been obtained from Guilin Medical College Affiliated Hospital Human Research Ethics Committee.

### Patient selection

During the period January 2010 to August 2010, patients meeting the following criteria were recruited from the Department of Radiation Oncology at the Affiliated Hospital of Guilin Medical University, Guangxi Province, China, which is located in southern China and provides health services for about 8 million people. Eligibility criteria included: patients who 1) were over 18 years, 2) were diagnosed with malignant tumor proven by biopsy, 3) would undergo RT with curative intent. Excluded from the study were patients who 1) had difficulty in understanding the questionnaire or communicating, 2) had a history of psychiatric disorder, 3) had distant metastasis. None of the patients died of RT-induced complications. Cancers were classified according to the 7th edition of the tumor-node-metastasis (TNM) staging system by the American Joint Committee on Cancer (AJCC)/ Union for International Cancer Control (UICC) [21]. Informed written consent from patients was obtained for the study.

### Randomization

All patients were randomly assigned to an intervention or control group. The computer-generated random allocation sequence was obtained independently by the investigators. Research nurses randomized participants 1:1 to either the control or intervention groups based on allocations before the RT.

### Psychosocial intervention

The psychosocial intervention was delivered by three conductors (a clinician, a nurse and a radiation therapist) who were trained in psychotherapy techniques. Each of these people had clinical experience in RT. All participants were given a series of questionnaires to complete.

The patients randomized to the intervention group of the study received psychosocial interventions according to their meeting problems included symptoms and side effects of treatment (i.e. fatigue, nausea, vomiting and pain), and psychological issues (i.e. depression, assessed by the Zung Self-rating Depression Scale [SDS]; and anxiety, assessed by the Self-rating Anxiety Scale [SAS]).

#### (1) Psychoeducation

Conductors should (a) show a good medical ethic and kindly attitude to patients, which could increase safe and confident feeling, and relieve their nervous tension to make them cooperate with treatment; (b) explain the necessity of tumor radiotherapy and introduce its principles, methods, adverse reactions during radiotherapy, prevention methods and treatment for side effects, so that patients may have some knowledge of radiotherapy; (c) analyze a variety of favorable factors with patients and their relatives together, encourage patients to maintain an optimistic mood, establish the confidence to conquer disease, and cooperate with the treatment; (d) enable patients to be familiar with therapeutic environment and equipment through visiting the radiotherapy room, and understanding the radiation process before the treatment, with the aim of reducing their fear and stress; (d) ask patients to carry out some wholesome activities during treatment, such as walking, listening to music, taking morning exercise, and so on, according to the patient’s psychological characteristics, education level and hobbies, to relieve panic and nervous tension; (e) illustrate the importance of companionship and comfort to the patient’s family, and strive for their cooperation, to enhance patients’ confidence in the treatment and returning to society. The session was carried out by a clinician, a nurse and a radiation therapist together.

#### (2) Cognitive-behavioral therapy (CBT)

A CBT protocol was offered based on recommendations in the literature [22-24]. In the first stage (1–2 weeks), through the above psychological education, conductors can build a good relationship with patients, have an insight into patients’ living environment, coping ability, coping styles, expectations, goals and so on, and discover any unreasonable cognitive concepts and attribution styles. In the second stage (2–3 weeks), conductors should look for facts and examples to correct patients’ unreasonable cognitive concepts and attribution styles for events, and reconstruct reasonable thoughts and a positive attribution style. In the third stage (3–4 weeks), conductors can use encouragement and a behavior-strengthening approach to consolidate the treatment effect, enable patients to have a better understanding of their attribution style through role playing, self-direction and so on, and strengthen the positive attribution style. The session was carried out by a clinician and a nurse.

#### (3) Supportive–expressive therapy

The goals of the supportive–expressive therapy was to create a supportive environment in which patients were helped to face their problems, fortify their relationships and explore positive meaning in their lives [25]. The principles of supportive–expressive therapy followed a treatment manual that had been well-established in cancer populations in previous reports [26]. Therapists provided a comfortable and safe environment to encourage participants to communicate their thoughts and feelings directly and openly with others, and promoted family and social contacts to further understand specific feelings (e.g. fear and grief) from patients with cancer, which could aid in obtaining more support, and cope with the threat of malignant tumor. The session was carried out by a clinician and a nurse.

The psychological intervention was provided in small cohorts ranging from 5 to 8 patients, and comprised two 60-min face-to-face interviews each week. In total, 8–12 therapy hours were delivered during RT.

Patients in the control group of the study received the usual education and medication from their therapist coordinator, in addition to RT.

### Outcome measures

#### Anxiety and depression

The primary endpoint will be the level of anxiety and depression as assessed by the SAS [27] and SDS [28], respectively. Each of the two scales is a self-completion questionnaire that has 20 items rating the four common characteristics of depression and anxiety. Scores for each question range from 1 to 4. The scores were counted up and multiplied by 1.25 to reach a standardized score, according to the instructions that accompany the scales. These questionnaires have previously been used in the Chinese population [29,30]. In accordance with the Chinese norm, a score of 50 or more on the SAS categorized individuals as having anxiety, and a score of more than 53 on the SDS categorized individuals as having depression. Higher scores indicate a greater psychological morbidity.

### Quality of life

Secondary endpoints will include QOL and overall survival.

QOL was assessed with the European Organization for Research and Treatment of Cancer Quality of Life Questionnaire-Core 30 (EORTC QLQ-C30), which is a reliable and valid instrument that is widely used for measuring QOL in cancer patients. EORTC QLQ-C30 comprises 30 questions related to the functioning and symptoms of cancer patients [31,32], including five functional scales (physical, role, cognitive, emotional and social), three symptom scales (fatigue, pain, and nausea and vomiting), one global health and QOL scale, and six symptom items (dyspnea,insomnia, appetite loss, constipation, diarrhea and financial difficulties).The scores of the items range from 0 to 100. The Chinese version of the EORTC QLQ-C30 has been validated [33-35]. Higher scores indicate better QOL for the functional scales and the global health scale, whereas higher scores indicate worse health for the symptom scales and items.

### Overall survival and follow-up

Overall survival (OS) was measured as the time from the date of randomization to death or the date of the last observed follow-up. Disease-free survival (DFS) was measured as the time from the date of randomization to the date of an event or the last follow-up date, where an ‘event’ is defined as recurrence, metastasis and death due to any cause. The follow-up information was recorded by the clinicians. Follow-up visits for OS and DFS occurred every 3 months for up to 2 years; the latest follow-up data were gathered in August 2012. The median follow-up period for the entire group was 25 months (range, 5–32 months).

### Statistical analysis

#### Sample size

To establish the sample size for the study, a 10% difference in scores for the outcome measure assessed by questionnaires between the two arms of the study was considered clinically meaningful and assuming a standard deviation of 10 [36]. With 65 patients in each group based on power calculations, there would be 80% power to detect a difference in scores at a 5% significance level. Each of the secondary outcomes is also powered at this level. Because of the poor prognosis for cancer patient we need to add our enrolment by 30% to account for attrition. Therefore, in total we will aim to recruit 170 patients.

#### Statistical analyses

Clinical and demographic variables were recorded in appropriate categories, and differences between the intervention group and the control group were assessed using chi-square tests or Fisher’s exact test. Student’s T test was used to compare sample means for study variables (anxiety, depression and QOL). OS was analyzed using Kaplan Meier survival analyses, and compared with the two-sided log-rank test; a two-sided *p* value < 0.05 was considered statistically significant. The statistical analyses were conducted with the software packages of SPSS (version 13.0).

## Results

### Patient characteristics

A total of 187 patients were consecutively enrolled in the trial. Of those, 6 patients were unable to complete the questionnaires because of aggravation of their health condition, and a further 3 patients could not be scheduled because of communication problems. The remaining 178 patients completed the assessments at baseline; the average age of the patients was 47 years, and those of women were 45 years and men were 48 years, respectively. All patients were randomly assigned to an individually tailored intervention (n = 89) or control (n = 89) group. The distribution of the major patient characteristics, treatment modalities and RT parameters was comparable for two groups′ participants (Table 1). None of the patient characteristics listed in Table 1 was significantly different at the 0.05 level between two groups.

**Table 1:** Sociodemographic and clinical characteristics of the patients

**Characteristic**	**Total n = 178(%)**	**IG n = 89(%)**	**CON n = 89(%)**	***p*value**
**Sex**				
**Male**	**75(42%)**	**38(43%)**	**37(42%)**	**0.879**
**Female**	**103(58%)**	**51(57%)**	**52(58%)**	
**Age (years)**				
**20-49**	**113(63%)**	**56(63%)**	**57(64%)**	**0.585**
**50-69**	**64(36%)**	**33(37%)**	**31(35%)**	
**70-79**	**1(1%)**	**0**	**1(1%)**	
**Marital status**				
**Married**	**172(97%)**	**87(98%)**	**85(96%)**	**0.406**
**Single/divorced/widow**	**6(3%)**	**2(2%)**	**4(4%)**	
**Education level**				
**Primary school**	**5(3%)**	**2(2%)**	**3(3%)**	**0.808**
**Junior high school**	**18(10%)**	**8(9%)**	**10(11%)**	
**Senior high school**	**86(48%)**	**46(52%)**	**40(45%)**	
**Above college**	**69(39%)**	**33(37%)**	**36(41%)**	
**Employment status**				
**Employed**	**131(73%)**	**67(75%)**	**64(72%)**	**0.707**
**Resigned**	**19(11%)**	**10(11%)**	**9(10%)**	
**Unemployed**	**28(16%)**	**12(13%)**	**16(18%)**	
**Locality**				
**Urban**	**78(44%)**	**40(45%)**	**38(43%)**	**0.763**
**Rural**	**100(56%)**	**49(55%)**	**51(57%)**	
**Primary cancer site**				
**Nasopharyngeal**	**62(35%)**	**31(35%)**	**31(35%)**	**0.989**
**Breast**	**41(23%)**	**19(21%)**	**22(25%)**	
**Gynecological cancer (cervical and endometrial)**	**31(17%)**	**14(16%)**	**17(19%)**	
**Lung**	**14(7%)**	**7(8%)**	**7(8%)**	
**Others (rectum\lymphoma\glioma etc.)**	**30(17%)**	**15(17%)**	**15(17%)**	
**ECOG performance status**				
**0 ~ 1**	**170(96%)**	**86(97%)**	**84(94%)**	**0.469**
**2**	**8(4%)**	**3(3%)**	**5(6%)**	
**Disease progression**				
**Advanced**	**111(62%)**	**53(60%)**	**58(65%)**	**0.439**
**Early stage**	**67(38%)**	**36(40%)**	**31(35%)**	
**History of operation**				
**Yes**	**87(49%)**	**44(49%)**	**43(48%)**	**0.881**
**No**	**91(51%)**	**45(51%)**	**46(52%)**	
**Radiotherapy dose**				
**≥70Gy**	**78(44%)**	**39(44%)**	**39(44%)**	**0.894**
**50 ~ 70Gy**	**97(54%)**	**49(55%)**	**48(54%)**	
**<50Gy**	**3(1%)**	**1(1%)**	**2(2%)**	
**Brachytherapy**	**14(7%)**	**6(7%)**	**8(9%)**	
**Chemotherapy**				
**None**	**52(29%)**	**29(33%)**	**23(26%)**	**0.710**
**Neoadjuvant**	**13(7%)**	**6(7%)**	**7(8%)**	
**Concomitant**	**77(43%)**	**35(39%)**	**42(47%)**	
**Adjuvant**	**102(57%)**	**50(56%)**	**52(58%)**	
**Comorbid condition**				
**None**	**158(89%)**	**77(87%)**	**81(91%)**	**0.877**
**Diabetes**	**7(4%)**	**4(4%)**	**3(3%)**	
**Hypertension**	**14(8%)**	**8(9%)**	**6(7%)**	
**Coronary heart disease**	**3(2%)**	**1(1%)**	**2(2%)**	
**Hepatitis B**	**3(2%)**	**2(2%)**	**1(1%)**	

Abbreviations: *IG* intervention group, *CON* control group.

### Main outcome measure

#### Anxiety and depression in patients

At baseline, a high proportion of enrolled patients were affected by anxiety (52%) and depression (48%), as assessed by SAS and SDS, respectively. Of these patients, women suffered from more anxiety (61%) and depression (53%) than men (anxiety, 39%; depression, 38%) (see Additional file 1). Female patients had a higher level of anxiety and depression compared with male patients, and differences were statistically significant. (see Additional file 2). Before radiation treatment, mean anxiety scores were 53.73 (SD = 11.88) and mean depression scores were 55.44 (SD = 9.18) in the intervention group, versus 52.63 (SD = 9.21) and 54.53 (SD = 8.30) in the control group, respectively. No significant variation in the scores of patients with anxiety (*p* = 0.492) and depression (*p* = 0.489) in the two arms was found before randomization. After RT, patients with anxiety (55.69, SD = 10.01) and depression (59.05, SD = 9.41) in the control group had significantly higher scores than patients with anxiety (48.78, SD = 8.95) and depression (51.48, SD = 7.54) in the intervention group (*p* < 0.001). Compared to control group patients, patients who had received a psychosocial intervention showed significantly lower scores of anxiety and depression (*p* < 0.001) after RT (Table 2).

**Table 2:** Comparisons of SAS, SDS at the baseline and 2 weeks post-RT between two groups (n = 178)

	**Baseline**				**2 weeks post-RT**			
	**IG (n = 89)**	**CON (n = 89)**			**IG (n = 89)**	**CON (n = 89)**		
	**Mean(SD)**	**Mean(SD)**	**t value**	***p*value**	**Mean(SD)**	**Mean(SD)**	**t value**	***p*value**
**SAS scores**	**53.73(11.88)**	**52.63(9.21)**	**0.689**	**0.492**	**48.78(8.95)**	**55.69(10.01)**	**−4.85**	**<0.001**
**SDS scores**	**55.44(9.18)**	**54.53(8.30)**	**0.693**	**0.489**	**51.48(7.54)**	**59.05(9.41)**	**−5.92**	**<0.001**

Abbreviations: *IG* intervention group, *CON* control group, *SD* standard deviation; RT, radiation treatment, *SAS* Self-rating Anxiety Scale, *SDS* Self-rating Depression Scale.

With stratified analysis, the data shown that the enhancement of psychological wellbeing of female patients were more significant than male patients (*p* < 0.001) (see Additional file 3). In addition, psychological symptoms with patients underwent adjuvant chemotherapy were improved obviously compared with the other chemotherapeutic groups (*p* < 0.001). (see Additional file 4).

### Secondary outcomes

#### Quality of life in patients

The mean QOL scores of the EORTC QLQ-C30 subscale before and after RT are shown in Table 3; higher scores reflect better QOL. Most of the subscale measures tended to become worse in the survey period. The decrease in QOL from baseline to post-RT was greater in the control arm, although the difference was not significant.

**Table 3:** Comparisons of QOL at the baseline and 2 weeks post-RT between the two groups (n = 178)

**EORTC QLQ-C30 subscales**	**Item**	**Baseline**				**2 weeks post-RT**			
		**IG (n = 89)**	**CON (n = 89)**			**IG (n = 89)**	**CON (n = 89)**		
		**Mean(SD)**	**Mean(SD)**	***t*value**	***p*value**	**Mean(SD)**	**Mean(SD)**	***t*value**	*p*value*
**Functioning scales**									
Physical functioning **PF**	**1-5**	77.23(10.19)	78.91(10.81)	−1.067	0.288	79.70(9.80)	75.36(9.71)	2.970	**0.003**
Role functioning **RF**	**6,7**	59.55(24.48)	58.28(24.49)	0.347	0.729	59.18(24.23)	57.45(21.01)	0.507	0.613
Emotional functioning **EF**	**21-24**	72.57(12.51)	70.77(14.71)	0.874	0.383	74.18(11.43)	66.03(14.00)	4.251	**<0.001**
Cognitive functioning **CF**	**20,25**	80.71(11.48)	81.27(11.46)	−0.327	0.744	81.09(12.36)	78.58(10.01)	1.489	0.138
Social functioning **SF**	**26,27**	75.00(13.82)	73.43(12.31)	0.802	0.424	74.35(10.67)	71.33(11.88)	1.782	0.076
Global health status **QL**	**29,30**	61.31(13.23)	58.43(12.78)	1.48	0.141	58.80(12.20)	51.12(12.82)	4.092	**<0.001**
**Symptom scales and/or items**									
Fatigue **FA**	**10,12,18**	28.05(14.75)	28.63(13.38)	−0.276	0.783	29.20(11.86)	32.93(13.78)	−1.938	0.054
Nausea/vomiting **NV**	**14,15**	11.05(11.50)	9.74(9.99)	0.812	0.418	14.61(12.26)	15.92(10.93)	−0.753	0.452
Pain **PA**	**9,19**	31.46(13.86)	30.52(14.70)	0.437	0.662	28.84(15.64)	30.90(16.77)	−0.847	0.398
Dyspnea **DY**	**8**	12.36(16.19)	14.79(16.56)	−0.992	0.323	11.98(16.08)	12.36(16.95)	−0.151	0.880
Insomnia **SL**	**11**	29.21(22.37)	30.71(21.45)	−0.456	0.649	27.71(20.25)	34.08(20.71)	−2.074	**0.040**
Appetite loss **AP**	**13**	23.03(22.96)	25.09(22.63)	−0.603	0.547	26.78(21.26)	28.84(23.13)	−0.618	0.537
Constipation **CO**	**16**	16.85(20.18)	16.10(20.17)	0.248	0.805	18.73(19.43)	20.97(19.71)	−0.766	0.445
Diarrhea **DI**	**17**	10.86(19.32)	9.36(15.88)	0.565	0.573	13.11(17.13)	12.36(18.38)	0.281	0.779
Financial difficulties **FI**	**28**	62.17(29.38)	61.42(29.26)	0.170	0.865	68.17(30.11)	67.04(28.65)	0.255	0.799

Abbreviations: *IG* intervention group, *CON* control group, *SD* standard deviation, *RT* radiation treatment; *EORTC QLQ-C30* European Organization for Research and Treatment of Cancer Quality of Life Questionnaire Core 30 items.

* *p* values in boldface indicate statistically significant difference.

Before the start of RT, there were few significant differences between the scores of the intervention and control groups. However, compared the QOL between women and men, we found that women always had lower QOL scores than men in our sample. Such as the scores of physical functioning (PF), emotional functioning (EF), cognitive functioning (CF), global health status (QL), etc. in females, were worse than those of males (p < 0.05) (see Additional file 5). After completion of RT, the two groups showed statistically significant differences in terms of global health status (*p* < 0.001). In relation to functional scales, physical functioning and emotional functioning scores were higher in the intervention group than in the control group (*p* < 0.01). In relation to symptom scales/items, significant improvement was seen in insomnia (*p* = 0.04) in the intervention group. Other items scores on the EORTC QOL instrument in the intervention arm indicated a trend towards improvement in comparison with the control arm. However, none of these changes attained statistical significance. Financial difficulties scores changed (but not significantly) in patients before and after RT (Table 3).

According to subgroup analysis, the result indicated that the improvement of QOL (i.e. PE, EF, QL, etc.) in female patients were more remarkable than male patients (see Additional file 6). Also, QOL of patients who received high dose radiotherapy (see Additional file 7) or underwent adjuvant chemotherapy (see Additional file 8) was significantly superior to that in the low dose radiotherapy group or other chemotherapeutic groups.

### Psychosocial intervention and survival

**Figure 2:**
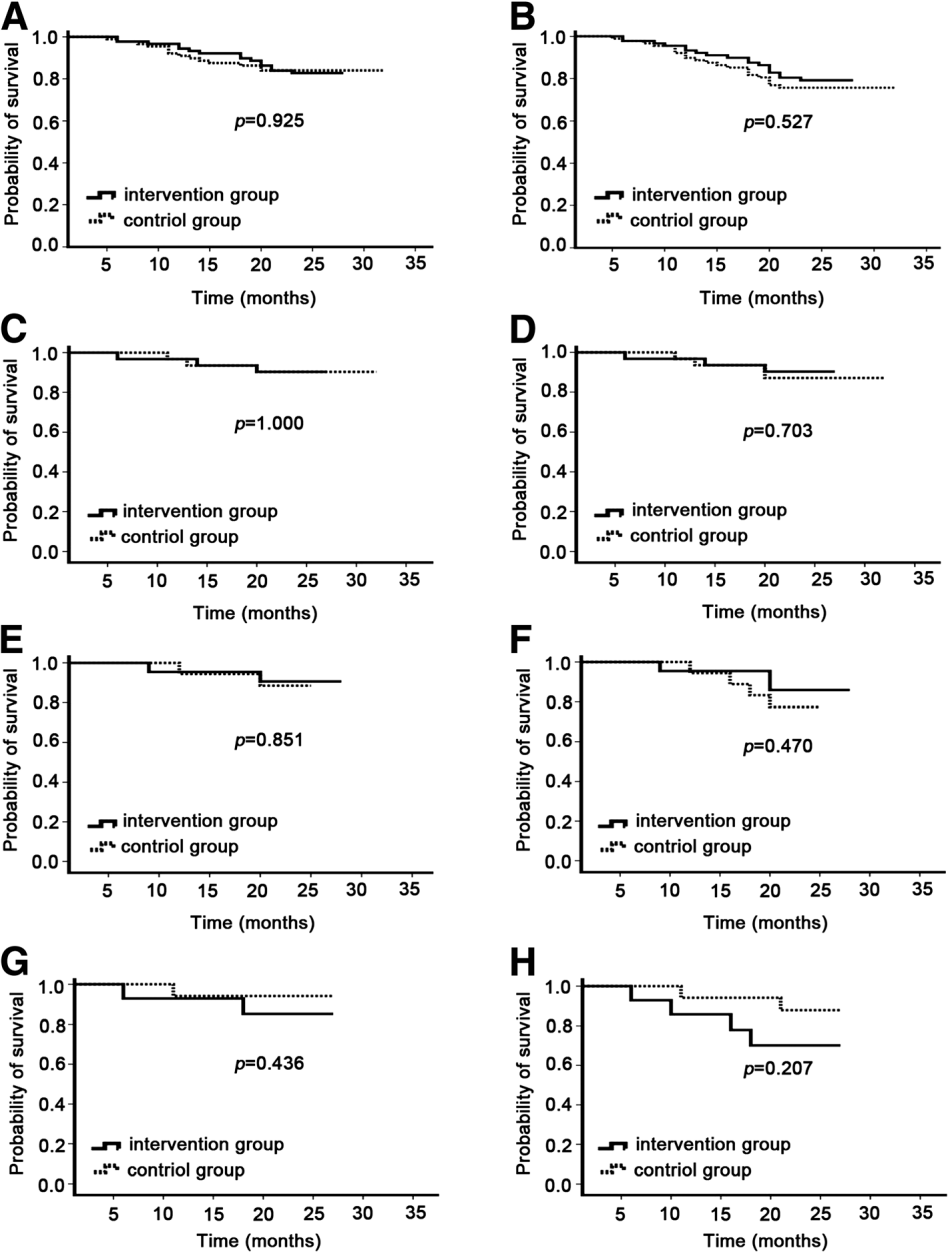
**Survival analysis in patients randomly assigned to intervention group or control group.** Kaplan–Meier survival curve for the overall survival in all patients **(A)**, nasopharyngeal carcinoma **(C)**, breast cancer **(E)** and gynecological cancer **(G)**. Kaplan–Meier survival curve for the time to progression in all patients **(B)**, nasopharyngeal carcinoma **(D)**, breast cancer **(F)** and gynecological cancer **(H)**.

The main OS and DFS analysis included the 178 patients. A total of 29 patients (16%) died – 15 (17%) in the intervention arm and 16 (18%) in the control arm; 10 patients (6%) were lost to follow-up – 4 (4%) in the intervention arm and 6 (7%) in the control arm. Disease-free survival rates at 2 years were 79.8% for the intervention arm and 76.4% for the control arm (two-sided log-rank, *p* = 0.527; Figure 2A). The 2-year overall survival rates were 83.1% for the intervention arm and 84.3% for the control arm (two-sided log-rank, *p* = 0.925; Figure 2B). At 2 years follow-up, there was no improvement in DFS and OS rate in the intervention arm compared with the control arm.

In the subgroup analysis, life table estimates of 2-year DFS for intervention group and control group were 90.3% and 87.1% (two-sided log-rank, *p* = 0.703) in nasopharyngeal carcinoma patients, 85.9% and 77.4% (two-sided log-rank, *p* = 0.470) in breast cancer patients, 70.1% and 87.8% (two-sided log-rank, *p* = 0.207) in gynecological cancer patients, respectively. 2-year OS were 90.3% and 90.3% (two-sided log-rank, *p* = 1.000) in nasopharyngeal carcinoma patients, 90.7% and 88.5% (two-sided log-rank, *p* = 0.851) in breast cancer patients, 85.1% and 94.1% (two-sided log-rank, *p* = 0.436) in gynecological cancer patients, respectively (Figure 2C-H).

## Discussion

The results of this randomized trial demonstrate that a psychosocial intervention significantly reduced levels of depression and anxiety compared to a control group. Further, the intervention was effective for improving elements of QOL, such as global health status and physical functioning; it also increased emotional functioning, significantly decreased insomnia, and was similar in cost-effectiveness in comparison with usual care. The subgroup analysis suggested that female patients and patients received high dose radiotherapy or underwent adjuvant chemotherapy would benefit more from the intervention. However, the intervention was not effective in prolonging survival.

More than half of the patients in our study undergoing RT for the various types of cancer expressed symptoms of depression or anxiety. This prevalence is consistent with previous reports of symptoms of psychosocial problems that ranged between 30% and 70% [37,38]. There were significant gender differences between anxiety and depression in our sample, and the female patients were generally more anxious and depressed compared to male patients. This result complied with Massie’s findings [39]. The reason for the appearance of negative moods in participants could be due to trepidation about a poor prognosis of cancer, misunderstanding of RT and worry about adverse effects of RT. In particular, for women with cancers, more misgivings were here compared to men, including fear of the diseases would impact their attractiveness, sexual relationships, fertility and even family happiness [40,41]. Despite a high prevalence of mental ill-health following the diagnosis of cancer, little effort has been applied to meeting such needs [42,43]. Therefore, it is imperative to assess the mental health of patients and take some measures to alleviate anxiety and depression throughout the process of RT.

The results of a psychosocial intervention in this study were encouraging. Following the psychosocial intervention, significant differences in depression and anxiety between two groups were observed after the completion of RT. A marked decrease in levels of depression and anxiety occurred within the intervention group. However, in the control group the results showed a trend for a deterioration. Thus, we could speculate that daily anticancer treatment will aggravate psychiatric distress if the patient does not also receive psychological care and provision of support from medical personnel during RT. The findings were in line with those reported in other studies. For example, Goerling U et al., using random sample analyses, reported an increase in the psychological condition of patients with cancer on a surgical ward after patients underwent psycho-oncological support [44]. Faul LA et al. designed a randomised study to evaluate the effectiveness of skill in stress management for cancer patients receiving chemotherapy. The authors demonstrated that psychosocial care was an efficient approach that clearly reduced anxiety and depression in patients with cancer [45]. Arguably, it is acceptable to define tailored psychological support plans whenever needed, with the aim of preventing or managing emotional problems appearing in RT.

This study also evaluated differences in QOL in a large sample of cancer patients, with and without intervention during RT. The results showed a trend for a deterioration in the control group compared to a stabilization in the intervention group. After RT, patients in the intervention group achieved significantly higher scores for global health status, physical and emotional functioning, and improvement in insomnia than patients in the control group. Therefore interventions are more necessary for these patients. Our findings agree with those from a randomized controlled trial conducted by Breitbart W, who found that participants who received psychotherapy demonstrated significantly greater improvement than the control group in terms of spiritual well-being and QOL [46]. Similarly, Eom CS et al. investigated the association between mental health, QOL and perceived social support in 1930 patients with cancer recruited from multiple centers and found that interventions improved mental health and QOL in cancer patients through a direct effect [47]. Moreover, our data showed that there was no significant difference in the financial difficulties subscale between the intervention and control groups at assessment after RT. Mean costs in the intervention group were CNY45,000, and were not higher than for the control group receiving usual care. The findings were similar to previous studies [48], and suggested that a psychological support during RT could be a cost-effective tool for improving QOL in patients with cancer.

Unplanned subgroup analysis demonstrated that obvious associations between psychological distress or QOL and clinical characteristics (including gender, radiation dose and chemotherapy modes) among oncology patients. A possible reason may be that women possessed more misgivings and trepidation, and high dose radiotherapy or adjuvant chemotherapy always resulted in severely toxic side effect, which significantly impacts mental status and QOL of patients. Thus, when screening of mental health and QOL in cancer patients who undergoing radiotherapy, female patients, those that received high dose radiotherapy, and those that underwent adjuvant chemotherapy should be routinely emphasized, who would benefit more from the psychosocial intervention.

Survival analyses from this trial indicated that patients with cancer randomized to receive a psychosocial intervention had no reduction in their risk for cancer recurrence and death compared to those who did not receive the intervention. An earlier finding that intervention was correlated with longer survival was not replicated [20]. Our results are in accordance with the literature showing that interventions for patients with cancer did not extended survival times [26,49,50]. Our results demonstrated that beneficial effects on survival is the major reason for patients receiving more anti-cancer treatment, and the key benefit of psychotherapeutic interventions is improved psychological well-being. However, the survival debate continues.

There is no consensus on how to define the psychological symptoms and problems of QOL in patients with cancer, and therapists always find it difficult to know what tests to order and which patients to treat, when and how long to treat, and what the available treatment options are. During the therapeutic process in our study, we observed that many factors could affect the moods and QOL, and that different patients varied in their receptiveness to psychosocial interventions. Although some of these factors cannot be avoided, psychosocial intervention effectively achieved benefits for patients. Therefore, as Simon Wein has stated, it is essential that clinical staff in oncology departments gain some knowledge of psycho-oncology, including communication skills, psychotropic medications and psychological therapies in routine clinical practice, so that they can clinically identify distress and provide initial psychosocial support if necessary [51].

Our trail has some potential limitations. First, duration of time of the survey for anxiety, depression and QOL was short, which meant that we were unable to determine what changes of moods and QOL in cancer patients would take place in the time after the end of RT. Second, survival following RT is often short, so the results may not exactly reflect the relationship between intervention therapy and survival. Finally, this study was performed using a single-center design and the sample size was relatively small, which may cause potential sampling errors. To investigate the usefulness and feasibility of intervention, further work, including a prospective longitudinal multicenter study, is recommended.

## Conclusion

With the use of new RT techniques (such as intensity-modulated radiation therapy [IMRT]) and multimodal treatment regimes in oncology practice, an increase in survival rates over the last few decades [52] had led to a greater proportion of patients calling for higher QOL after cancer treatment. The effect of individuals’ psychological well-being have become relevant parameters in oncology research and practice. The results of this randomized trial demonstrated that a psychosocial intervention during RT for patients with cancer is a practical, cost-effective tool for helping most patients receiving RT to reduce anxiety, depression and improve their QOL. The intervention was easy to implement during RT in hospital using health professionals (a clinician, a nurse, a radiation therapist ) who had undergone simple training in psychotherapy techniques, and that the treatment input required was minimal, which facilitated uptake by patients with cancer. Overall, psychosocial care during RT, as an important cure strategy, should be carried out in routine clinical practice.

## Abbreviations

QOL: Quality of life; RT: Radiation therapy; SDS: The zung self-rating depression scale; SAS: The self-rating anxiety scale; EORTC QLQ-C30) (QLQ-C30[3.0]: The European Organization for research and treatment of cancer quality of life questionnare-core 30; TNM: Tumor-node-metastasis; AJCC: The American joint committee on cancer; UICC: Union for international cancer control; CBT: Cognitive-behavioral therapy; OS: Overall survival; DFS: Disease-free survival; IG: Intervention group; CON: Control group; SD: Standard deviation; ECOG: The Eastern Cooperative oncology group.

## Competing interests

The authors have no actual or potential conflicts of interest to declare.

## Authors’ contributions

ZG and HYT conceived the project, participated in its design and performed psychosocial intervention and led manuscript development; HL provided methodological guidance and intellectual comment for psychosocial intervention, and helped to draft the manuscript; SKT performed the statistical analysis and guided interpretation of results; KHF and YCH participated in its design and coordination; QB provided expertise on chemotherapy and helped to draft the manuscript; WJ directed the study, its design, performed psychosocial intervention, and drafted the manuscript. All authors read and approved the final manuscript.

## Authors’ information

First author: share first authorship Zhen Guo and Hua-ying Tang.

## Supplementary Material

Additional file 1: Table S1 The prevalence (%) of women and men suffering from anxiety and depression. Click here for file

Additional file 2: Table S2 Comparisons of SAS and SDS between women and men at the baseline (n=178). Click here for file

Additional file 3: Table S3 Comparisons of SAS and SDS at the baseline and 2 weeks post-RT in male and female patients for subanalysis (n=178). Click here for file

Additional file 4: Table S4 Comparisons of SAS and SDS at the baseline and 2 weeks post-RT in in different chemotherapy for subanalysis (n=178). Click here for file

Additional file 5: Table S5 Comparisons of QOL between women and men at the baseline (n=178). Click here for file

Additional file 6: Table S6 Comparisons of QOL at the baseline and 2 weeks post-RT in male and female patients for subanalysis (n=178). Click here for file

Additional file 7: Table S7 Comparisons of QOL at the baseline and 2 weeks post-RT in different dose of radiotherapy for subanalysis (n=178). Click here for file

Additional file 8: Table S8 Comparisons of QOL at the baseline and 2 weeks post-RT in different chemotherapy for subanalysis (n=178). Click here for file
