# Supplementary material for: The benefits of psychosocial interventions for cancer patients undergoing radiotherapy
Source: Health Qual Life Outcomes. 2013 Jul 17;11:121. doi: 10.1186/1477-7525-11-121 (PMC3721996; doi:10.1186/1477-7525-11-121)
Supplement: Additional file 1: Table S1 — The prevalence (%) of women and men suffering from anxiety and depression. [file 1477-7525-11-121-S1.doc]

**Additional file 1: Table S1:** The prevalence (%) of women and men suffering from anxiety and depression.

|  | Total  n=178(%) | **Male**  **n=75(%)** | **Female**  **n=103(%)** |
| --- | --- | --- | --- |
| **anxiety** | 92(52%) | 29(39%) | 63(61%) |
| **depression** | 85(48%) | 30(40%) | 55(53%) |
