# Supplementary material for: The benefits of psychosocial interventions for cancer patients undergoing radiotherapy
Source: Health Qual Life Outcomes. 2013 Jul 17;11:121. doi: 10.1186/1477-7525-11-121 (PMC3721996; doi:10.1186/1477-7525-11-121)
Supplement: Additional file 2: Table S2 — Comparisons of SAS and SDS between women and men at the baseline (n=178). [file 1477-7525-11-121-S2.doc]

**Additional file 2: Table S 2: Comparisons of SAS and SDS between women and men at the baseline (n=178).**

|  | **Male**  **(n=75)** | **Female**  **(n=103)** |  |
| --- | --- | --- | --- |
|  | Mean(SD) | Mean(SD) | *p* value |
| **SAS scores** | 51.29(10.81) | 54.56(10.30) | **0.042** |
| **SDS scores** | 53.29(8.90) | 56.22(8.38) | **0.026** |
