# Supplementary material for: The benefits of psychosocial interventions for cancer patients undergoing radiotherapy
Source: Health Qual Life Outcomes. 2013 Jul 17;11:121. doi: 10.1186/1477-7525-11-121 (PMC3721996; doi:10.1186/1477-7525-11-121)
Supplement: Additional file 3: Table S3 — Comparisons of SAS and SDS at the baseline and 2 weeks post-RT in male and female patients for subanalysis (n=178). [file 1477-7525-11-121-S3.doc]

**Additional file 3: Table S3:** Comparisons of SAS and SDS at the baseline and 2 weeks post-RT in male and female patients for subanalysis (n=178).

|  | **Baseline** | | | | | | |  | **2 weeks post-RT** | | | | | | |
| --- | --- | --- | --- | --- | --- | --- | --- | --- | --- | --- | --- | --- | --- | --- | --- |
|  | **Male** | |  |  | **Female** | |  |  | **Male** | |  |  | **Female** | |  |
|  | **IG**  **(n=38)** | **CON**  **(n=37)** |  |  | **IG**  **(n=51)** | **CON**  **(n=52)** |  |  | **IG**  **(n=38)** | **CON**  **(n=37)** |  |  | **IG**  **(n=51)** | **CON**  **(n=52)** |  |
|  | Mean  (SD) | Mean  (SD) | *p* value |  | Mean  (SD) | Mean  (SD) | *p* value |  | Mean  (SD) | Mean  (SD) | *p* value |  | Mean  (SD) | Mean  (SD) | *p* value |
| **SAS scores** | 51.23  (11.81) | 51.35  (9.84) | 0.962 |  | 55.59  (11.70) | 53.54  (8.72) | 0.315 |  | 48.55  (8.82) | 52.94  (9.36) | **0.040** |  | 48.95  (9.14) | 57.65  (10.09) | **0.000** |
| **SDS scores** | 53.94  (9.62) | 52.62  (8.17) | 0.525 |  | 56.55  (8.77) | 55.89  (8.06) | 0.690 |  | 51.46  (6.78) | 55.99  (8.79) | **0.015** |  | 51.49  (8.13) | 61.23  (9.30) | **0.000** |
