# Supplementary material for: The benefits of psychosocial interventions for cancer patients undergoing radiotherapy
Source: Health Qual Life Outcomes. 2013 Jul 17;11:121. doi: 10.1186/1477-7525-11-121 (PMC3721996; doi:10.1186/1477-7525-11-121)
Supplement: Additional file 4: Table S4 — Comparisons of SAS and SDS at the baseline and 2 weeks post-RT in in different chemotherapy for subanalysis (n=178). [file 1477-7525-11-121-S4.doc]

**Additional file 4: Table S4:** Comparisons of SAS and SDS at the baseline and 2 weeks post-RT in in different chemotherapy for subanalysis (n=178).

|  | **Baseline** | | | | | | | | | | | |  | **2 weeks post-RT** | | | | | | | | | | | |
| --- | --- | --- | --- | --- | --- | --- | --- | --- | --- | --- | --- | --- | --- | --- | --- | --- | --- | --- | --- | --- | --- | --- | --- | --- | --- |
|  | **None CT** | | | **NACT** | | | **CRT** | | | **ACT** | | |  | **None CT** | | | **NACT** | | | **CRT** | | | **ACT** | | |
|  | **IG**  **(n=29)** | **CON**  **(n=23)** |  | **IG**  **(n=6)** | **CON**  **(n=7)** |  | **IG**  **(n=35)** | **CON**  **(n=42)** |  | **IG**  **(n=50)** | **CON**  **(n=52)** |  |  | **IG**  **(n=29)** | **CON**  **(n=23)** |  | **IG**  **(n=6)** | **CON**  **(n=7)** |  | **IG**  **(n=35)** | **CON**  **(n=42)** |  | **IG**  **(n=50)** | **CON**  **(n=52)** |  |
|  | Mean  (SD) | Mean  (SD) | *p* | Mean  (SD) | Mean  (SD) | *p* | Mean  (SD) | Mean  (SD) | *p* | Mean  (SD) | Mean  (SD) | *p* |  | Mean  (SD) | Mean  (SD) | *p* | Mean  (SD) | Mean  (SD) | *p* | Mean  (SD) | Mean  (SD) | *p* | Mean  (SD) | Mean  (SD) | *p* |
| **SAS scores** | 51.87  (11.41) | 52.07  (8.73) | 0.946 | 65.79  (12.57) | 55.68  (13.61) | 0.194 | 56.00  (12.54) | 52.30  (9.76) | 0.151 | 55.26  (12.00) | 53.85  (9.74) | 0.517 |  | 49.17  (8.70) | 59.09  (9.25) | **0.000** | 48.25  (5.59) | 57.14  (12.86) | 0.146 | 50.39  (9.33) | 54.67  (10.73) | 0.069 | 48.51  (8.98) | 55.05  (10.84) | **0.001** |
| **SDS scores** | 55.34  (10.92) | 52.34  (7.94) | 0.274 | 60.38  (6.68) | 56.75  (4.50) | 0.269 | 56.14  (8.24) | 55.50  (8.21) | 0.734 | 55.93  (7.75) | 55.78  (8.91) | 0.930 |  | 51.84  (7.56) | 61.33  (7.72) | **0.000** | 53.96  (8.57) | 60.25  (11.05) | 0.282 | 52.39  (7.85) | 57.78  (9.29) | **0.008** | 50.47  (7.02) | 58.75  (10.29) | **0.000** |
