# Supplementary material for: The benefits of psychosocial interventions for cancer patients undergoing radiotherapy
Source: Health Qual Life Outcomes. 2013 Jul 17;11:121. doi: 10.1186/1477-7525-11-121 (PMC3721996; doi:10.1186/1477-7525-11-121)
Supplement: Additional file 5: Table S5 — Comparisons of QOL between women and men at the baseline (n=178). [file 1477-7525-11-121-S5.doc]

**Additional file 5: Table S 5:** Comparisons of QOL between women and men at the baseline (n=178).

| **EORTC QLQ-C30**  **subscales** | **item** | **Male**  **（n=75）** | **Female**  **(n=103)** |  |
| --- | --- | --- | --- | --- |
|  |  | Mean(SD) | Mean(SD) | *p* value |
| **Functioning scales** |  |  |  |  |
| Physical functioning  **PF** | **1-5** | 80.24（10.34） | 76.49（10.40） | **0.018** |
| Role functioning  **RF** | **6,7** | 56.93（24.05） | 60.36（24.70） | 0.357 |
| Emotional functioning  **EF** | **21-24** | 75.53（14.08） | 68.86（12.67） | **0.001** |
| Cognitive functioning  **CF** | **20,25** | 83.33（11.62） | 83.33（11.62） | **0.019** |
| Social functioning  **SF** | **26,27** | 73.09（12.96） | 75.03（13.16） | 0.329 |
| Global health status  **QL** | **29,30** | 62.56（13.65） | 57.91（12.29） | **0.019** |
| **Symptom scales and/or items** |  |  |  |  |
| Fatigue  **FA** | **10,12,18** | 25.85（12.96） | 30.15（14.58） | 0.043 |
| Nausea/vomiting  **NV** | **14,15** | 8.00（9.24） | 12.14（11.48） | **0.011** |
| Pain  **PA** | **9,19** | 32.00（15.20） | 30.26（13.56） | 0.423 |
| Dyspnea  **DY** | **8** | 11.11（15.82） | 15.37（16.61） | 0.087 |
| Insomnia  **SL** | **11** | 24.44（22.15） | 33.98（20.87） | **0.004** |
| Appetite loss  **AP** | **13** | 22.67（22.03） | 25.08（23.32） | 0.486 |
| Constipation  **CO** | **16** | 15.11（19.98） | 17.47（20.26） | 0.44 |
| Diarrhea  **DI** | **17** | 8.00（16.29） | 11.65（18.50） | 0.174 |
| Financial difficulties  **FI** | **28** | 62.67（28.45） | 61.17（29.92） | 0.736 |
