# Supplementary material for: The benefits of psychosocial interventions for cancer patients undergoing radiotherapy
Source: Health Qual Life Outcomes. 2013 Jul 17;11:121. doi: 10.1186/1477-7525-11-121 (PMC3721996; doi:10.1186/1477-7525-11-121)
Supplement: Additional file 6: Table S6 — Comparisons of QOL at the baseline and 2 weeks post-RT in male and female patients for subanalysis (n=178). [file 1477-7525-11-121-S6.doc]

**Additional file 6: Table S 6:** Comparisons of QOL at the baseline and 2 weeks post-RT in male and female patients for subanalysis (n=178).

| **EORTC QLQ-C30**  **subscales** | **item** | **Baseline** | | | | | | | |  | **2 weeks post-RT** | | | | | | | |
| --- | --- | --- | --- | --- | --- | --- | --- | --- | --- | --- | --- | --- | --- | --- | --- | --- | --- | --- |
|  |  | **Male** | | |  |  | **Female** | |  |  | **Male** | |  |  | **Female** | | |  |
|  |  | **IG**  **(n=38)** | | **CON**  **(n=37)** |  |  | **IG**  **(n=51)** | **CON**  **(n=52)** |  |  | **IG**  **(n=38)** | **CON**  **(n=37)** |  |  | **IG**  **(n=51)** | | **CON**  **(n=52)** |  |
|  |  | Mean  (SD) | Mean  (SD) | | *p*  value | | Mean  (SD) | Mean  (SD) | *p*  value |  | Mean  (SD) | Mean  (SD) | *p*  value | | Mean  (SD) | | Mean  (SD) | *p* value |
| **Functioning scales** |  |  |  | |  | |  |  |  |  |  |  |  | |  | |  |  |
| Physical functioning  **PF** | **1-5** | 77.72  (10.31) | 82.84  (9.83) | | 0.031 | | 76.86  (10.19) | 76.12  (10.69) | 0.717 |  | 79.12  (11.09) | 77.66  (10.33) | 0.556 | | 80.13  (8.82) | | 73.72  (9.00) | **0.000** |
| Role functioning  **RF** | **6,7** | 56.14  (24.94) | 57.75  (23.43) | | 0.774 | | 62.09  (24.06) | 58.65  (25.43) | 0.483 |  | 56.58  (26.72) | 54.59  (21.19) | 0.723 | | 61.11  (22.28) | | 59.49  (20.85) | 0.703 |
| Emotional functioning  **EF** | **21-24** | 75.88  (12.82) | 75.18  (15.44) | | 0.832 | | 70.10  (11.81) | 67.64  (13.47) | 0.327 |  | 74.78  (13.71) | 67.57  (13.47) | **0.024** | | 73.72  (9.51) | | 64.94  (14.40) | **0.000** |
| Cognitive functioning  **CF** | **20,25** | 82.46  (12.22) | 84.23  (11.07) | | 0.512 | | 79.41  (10.84) | 79.17  (11.37) | 0.911 |  | 84.21  (12.22) | 79.37  (10.59) | 0.071 | | 78.76  (12.05) | | 78.01  (9.64) | 0.729 |
| Social functioning  **SF** | **26,27** | 73.25  (14.12) | 72.93  (11.83) | | 0.916 | | 76.31  (13.58) | 73.78  (12.75) | 0.333 |  | 73.25  (10.64) | 69.55  (12.77) | 0.177 | | 75.16  (10.73) | 72.60  (11.15) | | 0.236 |
| Global health status  **QL** | **29,30** | 64.48  (13.79) | 60.59  (13.41) | | 0.220 | | 58.95  (12.41) | 56.89  (12.21) | 0.397 |  | 58.77  (12.40) | 48.20  (10.96) | **0.000** | | 58.82  (12.18) | 53.20  (13.72) | | **0.030** |
| **Symptom scales and/or items** |  |  |  | |  | |  |  |  |  |  |  |  | |  |  | |  |
| Fatigue  **FA** | **10,12,18** | 24.76  (11.68) | 26.97  (14.23) | | 0.464 | | 30.50  (16.36) | 29.81  (12.75) | 0.813 |  | 27.91  (12.70) | 31.06  (13.60) | 0.303 | | 30.15  (11.23) | 34.26  (13.88) | | 0.102 |
| Nausea/vomiting  **NV** | **14,15** | 7.46  (9.25) | 8.56  (9.32) | | 0.609 | | 13.73  (12.34) | 10.58  (10.45) | 0.165 |  | 14.91  (12.12) | 15.32  (10.67) | 0.879 | | 14.38  (12.48) | 16.35  (11.19) | | 0.401 |
| Pain  **PA** | **9,19** | 32.46  (13.39) | 31.53  (17.02) | | 0.794 | | 30.72  (14.29) | 29.81  (12.92) | 0.735 |  | 31.58  (16.34) | 33.78  (12.10) | 0.510 | | 26.80  (14.94) | 28.85  (19.28) | | 0.549 |
| Dyspnea  **DY** | **8** | 10.53  (15.70) | 11.71  (16.13) | | 0.748 | | 13.72  (16.57) | 16.99  (16.66) | 0.322 |  | 11.40  (16.02) | 9.91  (15.44) | 0.682 | | 12.42  (16.27) | 14.10  (17.89) | | 0.619 |
| Insomnia  **SL** | **11** | 23.68  (21.79) | 25.22  (22.78) | | 0.766 | | 33.33  (22.11) | 34.61  (19.76) | 0.757 |  | 29.82  (18.65) | 30.63  (19.84) | 0.857 | | 26.14  (21.41) | 36.54  (21.14) | | **0.015** |
| Appetite loss  **AP** | **13** | 21.05  (19.64) | 24.32  (24.40) | | 0.524 | | 24.51  (25.24) | 25.64  (21.51) | 0.807 |  | 32.45  (21.21) | 32.43  (25.44) | 0.996 | | 22.55  (20.50) | 26.28  (21.22) | | 0.366 |
| Constipation  **CO** | **16** | 14.03  (19.96) | 16.22  (20.22) | | 0.640 | | 18.95  (20.28) | 16.02  (20.33) | 0.466 |  | 19.30  (18.39) | 17.12  (18.63) | 0.611 | | 18.30  (20.34) | 23.72  (20.17) | | 0.178 |
| Diarrhea  **DI** | **17** | 7.89  (18.07) | 8.11  (14.50) | | 0.955 | | 13.07  (20.09) | 10.26  (16.88) | 0.443 |  | 13.16  (16.51) | 16.21  (18.63) | 0.454 | | 13.07  (17.74) | 9.61  (17.88) | | 0.327 |
| Financial difficulties  **FI** | **28** | 61.40  (25.15) | 63.96  (31.80) | | 0.700 | | 62.75  (32.42) | 59.62  (27.49) | 0.598 |  | 71.05  (28.13) | 66.67  (30.43) | 0.519 | | 66.01  (31.62) | 67.31  (27.61) | | 0.825 |
