# Supplementary material for: The benefits of psychosocial interventions for cancer patients undergoing radiotherapy
Source: Health Qual Life Outcomes. 2013 Jul 17;11:121. doi: 10.1186/1477-7525-11-121 (PMC3721996; doi:10.1186/1477-7525-11-121)
Supplement: Additional file 7: Table S7 — Comparisons of QOL at the baseline and 2 weeks post-RT in different dose of radiotherapy for subanalysis (n=178). [file 1477-7525-11-121-S7.doc]

**Additional file 7: Table S7: Comparisons of QOL at the baseline and 2 weeks post-RT in different dose of radiotherapy for subanalysis** (n=178).

| **EORTC QLQ-C30**  **subscales** | **item** | **Baseline** | | | | | | |  | **2 weeks post-RT** | | | | | | |
| --- | --- | --- | --- | --- | --- | --- | --- | --- | --- | --- | --- | --- | --- | --- | --- | --- |
|  |  | **≥70Gy** | |  |  | **70Gy<** | |  |  | **≥70Gy** | |  |  | **70Gy<** | |  |
|  |  | **IG**  **(n=39)** | **CON**  **(n=39)** |  |  | **IG**  **(n=50)** | **CON**  **(n=50)** |  |  | **IG**  **(n=39)** | **CON**  **(n=39)** |  |  | **IG**  **(n=50)** | **CON**  **(n=50)** |  |
|  |  | Mean  (SD) | Mean  (SD) | *p*  value | | Mean  (SD) | Mean  (SD) | *p*  value |  | Mean  (SD) | Mean  (SD) | *p*  value | | Mean  (SD) | Mean  (SD) | *p*  value |
| **Functioning scales** |  |  |  |  | |  |  |  |  |  |  |  | |  |  |  |
| Physical functioning  **PF** | **1-5** | 78.12  (8.78) | 78.98  (11.50) | 0.714 | | 76.53  (11.21) | 78.86  (10.37) | 0.284 |  | 81.03  (8.99) | 77.61  (11.60) | 0.150 | | 78.67  (10.37) | 73.60  (7.61) | **0.006** |
| Role functioning  **RF** | **6,7** | 62.39  (25.28) | 63.76  (25.35) | 0.812 | | 57.33  (23.85) | 54.00  (23.14) | 0.480 |  | 65.81  (19.85) | 63.16  (20.77) | 0.566 | | 54.00  (26.20) | 53.00  (20.30) | 0.831 |
| Emotional functioning  **EF** | **21-24** | 73.93  (11.81) | 70.08  (16.55) | 0.241 | | 71.50  (13.05) | 71.31  (13.27) | 0.944 |  | 78.76  (10.59) | 67.61  (13.15) | **0.000** | | 70.60  (10.87) | 64.80  (14.61) | **0.027** |
| Cognitive functioning  **CF** | **20,25** | 79.06  (11.29) | 81.63  (12.56) | 0.346 | | 82.00  (11.58) | 81.00  (10.65) | 0.654 |  | 84.61  (11.71) | 76.07  (10.54) | **0.001** | | 78.33  (12.25) | 80.53  (9.22) | 0.313 |
| Social functioning  **SF** | **26,27** | 76.50  (14.54) | 69.66  (14.32) | 0.040 | | 73.84  (13.26) | 76.37  (9.65) | 0.277 |  | 73.08  (10.55) | 65.68  (12.47) | **0.006** | | 75.33  (10.77) | 75.73  (9.36) | 0.844 |
| Global health status  **QL** | **29,30** | 61.33  (14.99) | 56.41  (14.24) | 0.142 | | 61.30  (11.84) | 60.00  (11.42) | 0.577 |  | 63.25  (11.43) | 46.80  (12.18) | **0.000** | | 55.33  (11.76) | 54.50  (12.40) | 0.731 |
| **Symptom scales and/or items** |  |  |  |  | |  |  |  |  |  |  |  | |  |  |  |
| Fatigue  **FA** | **10,12,18** | 30.53  (15.33) | 26.98  (11.37) | 0.249 | | 26.11  (14.14) | 29.92  (14.75) | 0.191 |  | 28.33  (10.90) | 35.59  (16.99) | **0.028** | | 29.87  (12.63) | 30.86  (10.34) | 0.670 |
| Nausea/vomiting  **NV** | **14,15** | 9.40  (10.68) | 8.98  (10.01) | 0.856 | | 12.34  (12.05) | 10.33  (10.04) | 0.369 |  | 14.10  (11.17) | 17.52  (9.32) | 0.146 | | 15.00  (13.15) | 14.67  (11.97) | 0.895 |
| Pain  **PA** | **9,19** | 31.63  (14.20) | 32.48  (16.20) | 0.805 | | 31.33  (13.73) | 29.00  (13.39) | 0.392 |  | 26.50  (12.52) | 32.05  (15.94) | 0.091 | | 30.67  (17.61) | 30.00  (17.50) | 0.850 |
| Dyspnea  **DY** | **8** | 11.11  (15.92) | 15.38  (16.83) | 0.253 | | 13.33  (16.49) | 14.33  (16.50) | 0.762 |  | 9.40  (15.19) | 11.11  (15.92) | 0.629 | | 14.00  (16.62) | 13.33  (17.82) | 0.847 |
| Insomnia  **SL** | **11** | 33.33  (21.63) | 34.19  (24.77) | 0.871 | | 26.00  (22.63) | 28.00  (18.27) | 0.628 |  | 28.20  (18.00) | 33.33  (20.23) | 0.241 | | 27.33  (22.02) | 34.67  (21.25) | 0.093 |
| Appetite loss  **AP** | **13** | 25.64  (20.89) | 26.49  (23.17) | 0.865 | | 21.00  (24.47) | 24.00  (22.38) | 0.524 |  | 27.35  (21.46) | 30.77  (24.64) | 0.515 | | 26.33  (21.32) | 27.33  (22.02) | 0.818 |
| Constipation  **CO** | **16** | 17.09  (22.78) | 17.95  (21.42) | 0.865 | | 16.67  (18.14) | 14.67  (19.24) | 0.594 |  | 16.24  (16.88) | 23.08  (21.84) | 0.126 | | 20.67  (21.18) | 19.33  (17.93) | 0.735 |
| Diarrhea  **DI** | **17** | 9.40  (20.16) | 8.55  (16.61) | 0.839 | | 12.00  (18.76) | 10.00  (15.43) | 0.562 |  | 11.11  (15.92) | 14.53  (19.93) | 0.405 | | 14.67  (18.02) | 10.67  (17.09) | 0.258 |
| Financial difficulties  **FI** | **28** | 57.27  (28.56) | 70.09  (30.39) | 0.059 | | 66.00  (29.73) | 54.67  (26.74) | 0.048 |  | 64.10  (30.00) | 72.65  (29.49) | 0.208 | | 71.33  (30.13) | 62.67  (27.47) | 0.136 |
