# Supplementary material for: The benefits of psychosocial interventions for cancer patients undergoing radiotherapy
Source: Health Qual Life Outcomes. 2013 Jul 17;11:121. doi: 10.1186/1477-7525-11-121 (PMC3721996; doi:10.1186/1477-7525-11-121)
Supplement: Additional file 8: Table S8 — Comparisons of QOL at the baseline and 2 weeks post-RT in different chemotherapy for subanalysis (n=178). [file 1477-7525-11-121-S8.doc]

**Additional file 8: Table S8:** Comparisons of QOL at the baseline and 2 weeks post-RT in different chemotherapy for subanalysis (n=178).

| **EORTC QLQ-C30**  **subscales** | **item** | **Baseline** | | | | | | | | | | | |  |  | **2 weeks post-RT** | | | | | | | | | | | | | |
| --- | --- | --- | --- | --- | --- | --- | --- | --- | --- | --- | --- | --- | --- | --- | --- | --- | --- | --- | --- | --- | --- | --- | --- | --- | --- | --- | --- | --- | --- |
|  |  | **None CT** | | | **NACT** | | | **CRT** | | | **ACT** | | | | **None CT** | | | | **NACT** | | | **CRT** | | | | **ACT** | | | |
|  |  | **IG**  **(n=29)** | **CON**  **(n=23)** |  | **IG**  **(n=6)** | **CON**  **(n=7)** |  | **IG**  **(n=35)** | **CON**  **(n=42)** |  | **IG**  **(n=50)** | **CON**  **(n=52)** |  | | **IG**  **(n=29)** | | **CON**  **(n=23)** |  | **IG**  **(n=6)** | **CON**  **(n=7)** |  | | **IG**  **(n=35)** | **CON**  **(n=42)** |  | | **IG**  **(n=50)** | **CON**  **(n=52)** |  |
|  |  | Mean  (SD) | Mean  (SD) | *p* | Mean  (SD) | Mean  (SD) | *p* | Mean  (SD) | Mean  (SD) | *p* | Mean  (SD) | Mean  (SD) | ***P*** | | Mean  (SD) | | Mean  (SD) | *p* | Mean  (SD) | Mean  (SD) | *p* | | Mean  (SD) | Mean  (SD) | *p* | | Mean  (SD) | Mean  (SD) | ***P*** |
| **Functioning scales** |  |  |  |  |  |  |  |  |  |  |  |  |  | |  | |  |  |  |  |  | |  |  |  | |  |  |  |
| Physical functioning  **PF** | **1-5** | 77.93  (11.70) | 78.26  (10.86) | 0.918 | 77.78  (9.11) | 71.43  (14.25) | 0.369 | 77.72  (9.00) | 78.53  (11.50) | 0.734 | 77.07  (9.44) | 79.55  (10.91) | 0.224 | | 77.70  (10.43) | | 74.78  (9.20) | 0.296 | 73.33  (8.43) | 68.57  (8.36) | 0.330 | | 78.10  (10.55) | 74.92  (11.48) | 0.214 | | 80.93  (8.76) | 76.28  (9.92) | **0.014** |
| Role functioning  **RF** | **6,7** | 56.32  (24.16) | 66.81  (20.75) | 0.105 | 47.22  (30.58) | 47.62  (17.82) | 0.977 | 60.95  (25.55) | 59.60  (26.05) | 0.82 | 63.33  (22.84) | 52.89  (24.34) | 0.028 | | 54.60  (27.42) | | 58.55  (26.01) | 0.600 | 44.44  (17.22) | 47.62  (15.00) | 0.729 | | 58.10  (20.36) | 59.60  (19.72) | 0.743 | | 62.67  (22.48) | 56.09  (20.20) | 0.123 |
| Emotional functioning  **EF** | **21-24** | 75.00  (9.71) | 74.78  (16.04) | 0.952 | 79.17  (6.97) | 62.62  (14.40) | 0.027 | 72.14  (14.28) | 68.77  (15.02) | 0.319 | 71.67  (11.42) | 70.62 (14.69) | 0.69 | | 71.67  (12.45) | | 67.39  (14.39) | 0.256 | 69.17  (6.56) | 66.43  (15.82) | 0.701 | | 75.90  (11.86) | 68.73  (12.69) | **0.013** | | 75.53  (10.04) | 63.49 (14.58) | **0.000** |
| Cognitive functioning  **CF** | **20,25** | 81.61  (13.62) | 81.16  (9.13) | 0.892 | 75.00  (9.13) | 76.19  (8.91) | 0.817 | 80.00  (10.54) | 80.16  (12.34) | 0.952 | 81.00  (10.65) | 82.69  (12.33) | 0.461 | | 81.61  (14.33) | | 78.26  (9.84) | 0.344 | 75.00  (9.13) | 77.62  (6.59) | 0.561 | | 80.00  (11.99) | 76.59  (10.24) | 0.182 | | 81.67  (11.29) | 79.36 (10.29) | 0.283 |
| Social functioning  **SF** | **26,27** | 70.98  (12.72) | 69.93  (9.02) | 0.74 | 80.56  (12.54) | 80.00  (9.23) | 0.928 | 76.67  (13.37) | 72.94  (15.05) | 0.259 | 76.67  (14.18) | 74.62 (12.95) | 0.447 | | 75.29  (12.30) | | 69.56  (10.79) | 0.085 | 77.79  (8.57) | (8.60) (6.34) | 0.852 | | 72.86  (8.18) | 70.59  (13.43) | 0.386 | | 74.33  (10.22) | 71.31 (12.28) | 0.181 |
| Global health status  **QL** | **29,30** | 61.49  (12.08) | 57.25  (12.13) | 0.215 | 61.11  (6.81) | 57.14  (16.27) | 0.59 | 61.67  (14.88) | 57.74  (13.57) | 0.229 | 61.97  (13.87) | 59.62  (13.24) | 0.383 | | 58.05  (12.69) | | 50.00  (13.05) | **0.029** | 52.78  (10.09) | 48.81  (13.97) | 0.575 | | 59.29  (11.39) | 47.42  (11.71) | **0.000** | | 59.33  (11.86) | 52.08  (13.09) | **0.004** |
| **Symptom scales and/or items** |  |  |  |  |  |  |  |  |  |  |  |  |  | |  | |  |  |  |  |  | |  |  |  | |  |  |  |
| Fatigue  **FA** | **10,12,18** | 26.31  (14.61) | 28.91  (14.87) | 0.53 | 27.67  (11.69) | 31.66  (13.49) | 0.583 | 30.24  (15.72) | 27.97  (12.10) | 0.476 | 28.47  (13.62) | 28.33  (13.46) | 0.956 | | 31.72  (11.78) | | 30.26  (11.26) | 0.652 | 38.83  (6.02) | 26.81  (8.80) | **0.017** | | 29.04  (10.98) | 35.16  (16.50) | 0.065 | | 27.58(11.68) | 34.26  (15.21) | **0.015** |
| Nausea/vomiting  **NV** | **14,15** | 11.50  (14.16) | 10.87  (11.90) | 0.866 | 13.89(12.55) | 11.91  (12.60) | 0.782 | 10.00  (10.85) | 9.92  (9.78) | 0.973 | 11.67  (10.24) | 8.66 (9.62) | 0.129 | | 14.94  (12.87) | | 13.05  (8.64) | 0.547 | 25.00  (9.13) | 14.29  (6.30) | **0.030** | | 16.67  (12.13) | 16.67  (11.04) | 1.000 | | 14.33  (11.67) | 17.95 (11.36) | 0.116 |
| Pain  **PA** | **9,19** | 33.33  (14.08) | 29.71  (15.86) | 0.388 | 38.89(20.18) | 30.95  (11.50) | 0.393 | 32.38  (13.37) | 31.75  (15.96) | 0.852 | 30.33  (13.75) | 29.17  (11.84) | 0.647 | | 33.91  (19.15) | | 33.33  (14.21) | 0.905 | 30.56  (12.54) | 16.67  (13.61) | 0.084 | | 29.05  (13.00) | 28.97  (15.64) | 0.981 | | 25.33  (12.70) | 30.77 (17.89) | 0.081 |
| Dyspnea  **DY** | **8** | 11.49  (16.12) | 18.11  (16.60) | 0.153 | 16.67  (18.26) | 14.28  (17.82) | 0.817 | 12.38  (16.34) | 15.87  (16.85) | 0.362 | 12.00  (16.16) | 12.82  (16.37) | 0.8 | | 11.49  (16.12) | | 8.69  (14.96) | 0.524 | 16.67  (8.26) | 9.52  (16.26) | 0.471 | | 12.38  (16.34) | 11.11  (15.90) | 0.731 | | 12.67  (16.34) | 14.74  (17.97) | 0.543 |
| Insomnia  **SL** | **11** | 26.44  (22.50) | 31.88  (21.27) | 0.379 | 27.78  (32.77) | 28.57  (12.60) | 0.954 | 30.47  (21.95) | 32.54  (23.84) | 0.696 | 30.00  (22.59) | 27.56  (19.49) | 0.561 | | 35.63  (19.78) | | 28.98  (18.27) | 0.219 | 27.78  (25.09) | 28.57  (12.60) | 0.943 | | 27.62  (18.94) | 35.71  (20.02) | 0.074 | | 23.33  (19.34) | 36.54  (21.14) | **0.001** |
| Appetite loss  **AP** | **13** | 21.26  (19.36) | 27.53  (25.92) | 0.323 | 33.33  (29.82) | 33.33  (3.33) | 1.000 | 24.77  (21.91) | 26.19  (22.73) | 0.781 | 24.00  (25.23) | 25.00  (21.77) | 0.831 | | 28.16  (23.19) | | 27.53  (23.89) | 0.924 | 27.78  (13.61) | 23.81  (25.20) | 0.738 | | 32.38  (18.94) | 30.16  (25.30) | 0.669 | | 23.33  (19.34) | 30.13  (24.04) | 0.120 |
| Constipation  **CO** | **16** | 11.49  (16.12) | 15.94  (17.02) | 0.34 | 22.22  (17.21) | 23.81  (16.26) | 0.867 | 20.95  (22.99) | 19.05  (22.26) | 0.714 | 16.67  (20.48) | 17.31 (22.38) | 0.88 | | 17.24  (19.15) | | 21.74  (19.09) | 0.404 | 27.78  (25.09) | 14.28  (17.82) | 0.282 | | 18.09  (18.69) | 21.43  (20.59) | 0.463 | | 18.67  (19.24) | 19.23 (19.07) | 0.882 |
| Diarrhea  **DI** | **17** | 12.64  (20.73) | 10.14  (15.6) | 0.634 | 11.11  (17.21) | 9.52  (16.26) | 0.867 | 9.52  (19.08) | 10.32  (17.25) | 0.849 | 10.67  (19.56) | 8.33 (16.00) | 0.51 | | 14.94  (19.08) | | 11.59  (19.09) | 0.533 | 11.11  (17.21) | 9.52  (16.26) | 0.867 | | 12.38  (16.34) | 16.67  (19.82) | 0.310 | | 4.00  (16.62) | 11.54  (18.53) | 0.482 |
| Financial difficulties  **FI** | **28** | 50.57  (29.03) | 60.87  (27.80) | 0.202 | 66.67  (29.82) | 57.14  (37.09) | 0.624 | 61.91  (25.75) | 65.87  (30.79) | 0.546 | 68.00  (29.32) | 59.62  (28.27) | 0.145 | | 64.37  (34.42) | | 66.67  (26.59) | 0.793 | 72.22  (25.09) | 66.67 (3.33) | 0.744 | | 65.72  (26.18) | 70.64  (31.41) | 0.463 | | 70.00  (28.77) | 66.67 (27.22) | 0.549 |
